# Adherence to Pharmacotherapy and Medication-Related Beliefs in Patients with Hypertension in Lima, Peru

**DOI:** 10.1371/journal.pone.0112875

**Published:** 2014-12-03

**Authors:** Marta Fernandez-Arias, Ana Acuna-Villaorduna, J. Jaime Miranda, Francisco Diez-Canseco, German Malaga

**Affiliations:** 1 CRONICAS Centre of Excellence in Chronic Diseases, Universidad Peruana Cayetano Heredia, Lima, Peru; 2 Global Health, Brighton and Sussex Medical School, Brighton, United Kingdom; 3 Unidad de Conocimiento y Evidencia, Universidad Peruana Cayetano Heredia, Lima, Peru; 4 Facultad de Medicina “Alberto Hurtado”, Universidad Peruana Cayetano Heredia, Lima, Peru; 5 Servicio de Medicina Interna, Hospital Nacional Cayetano Heredia, Lima, Perú; Hospital de Clínicas de Porto Alegre, Brazil

## Abstract

**Objective:**

To characterize adherence to pharmacological medication and beliefs towards medication in a group of patients with hypertension in a large national hospital.

**Materials and Methods:**

Cross-sectional survey among patients with hypertension attending the outpatient clinic of a large national hospital. Exposure of interest was the patient's beliefs towards general medication and antihypertensive drugs, i.e. beliefs of harm, overuse, necessity and concern, measured using the Beliefs about Medication questionnaire. Main outcome was adherence measured using the Morisky Medication Adherence Scale-8. Multivariate analysis was conducted using Poisson distribution logistic regression, prevalence ratios and 95% confidence intervals were calculated.

**Results:**

Data from 115 participants, 67% females and mean age 62.7 years were analyzed. Low adherence was found in 57.4%. Highest scores were on the ideas of necessity and one of the most rated statements was “physicians would prescribe less medication if they spent more time with patients”. Beliefs of harm about medications and concerns about antihypertensive drugs were higher in the low adherence group (p<0.01). Those who scored higher on ideas of harm were 52% less likely of being high adherents (PR 0.48; 95% CI 0.25–0.93) and those with higher scores on concerns were 41% less likely of being high adherents (PR 0.59; 95% CI 0.39–0.91). Patients whose ideas of necessity outweighed their concerns were more likely to be adherent (PR 2.65; 95% CI 1.21–5.81).

**Conclusions:**

Low adherence to antihypertensive medication is common. High scores on ideas of harm, concern and a high necessity-concern differential were predictors of medication adherence.

## Introduction

Hypertension is a well-recognized risk factor for the development of cardiovascular and cerebrovascular disease [1]. In 2008, it was estimated that 40% of the global population over the age of 25 had high blood pressure levels and that hypertension was related to 13% of the global death rate [2]. In Latin America, the burden of non-communicable diseases has led to an increase in the prevalence of hypertension and its related mortality which accounted for two thirds of all deaths by the year 2000, and thus establishing this disease as a public health concern in many geographic locations [1], [3], [4]. In Peru, it is considered the second cause of mortality and the eleventh cause of burden of disease with a prevalence ranging from 8% to 30% as reported by several local studies [4]–[6].

Despite the worldwide availability of antihypertensive therapy, which has shown to be effective in decreasing the risk of myocardial infarction and stroke among other benefits [7], only 25% of treated patients are able to achieve optimal blood pressure control and almost 50% quit their treatment within the first year [8]. The statistics are even more striking in developing countries such as Peru, where a study reported a rate of controlled hypertension of only 14% [6]. Several studies have shown that of the different factors contributing to poor blood pressure control, poor adherence is the primary one, which is also responsible for the decline in patients' quality of life and the squandering of health care system resources [9]–[11].

Adherence, defined as the extent to which a person's behavior corresponds with agreed recommendations from a health care provider [8], has been recognized as a health care problem; especially among patients suffering chronic diseases which imply the need of interventions for long periods of time [12], [13]. Certain barriers to adherence have been previously described as multifactorial. Some of them are related to the health system itself, which is difficult to modify, while others include those related to the patient's level of knowledge about the disease, as well as beliefs and attitudes towards medication, both general and specific to their conditions, which can be targeted by health care policies [12], [14], [15].

Some studies suggested that beliefs and perceptions regarding medication may have an important effect on adherence to treatment. In this way, increased positive beliefs such as necessity are linked to increased levels of adherence [23]. Thus, it is critical to have a better understanding about the patient's beliefs about medications that could translate into poor adherence in order to focus interventional efforts on improving them in the future. This study was aimed to characterize the level of adherence to pharmacological medication and beliefs towards medication usage in a group of patients with hypertension in a large Peruvian hospital.

## Materials and Methods

### Study Design

Cross-sectional survey conducted among patients with an established diagnosis of hypertension attending the outpatient clinics at Hospital Nacional Cayetano Heredia (HNCH) during May and June 2012.

### Setting and Participants

HNCH is a 420-bed tertiary level, university affiliated national hospital located in the northern area of Lima, Peru. It is the primary referral hospital for the area and serves a population of approximately 3.5 million, mainly from low-income groups. HNCH has been designated by Peru's Ministry of Health as part of the selected group of national specialized hospitals to lead on the management and care of individuals with chronic conditions [16].

Adult patients in the waiting rooms of the cardiology and endocrinology clinics that admitted to have a medical diagnosis of hypertension and to take at least one anti-hypertensive medication were selected by convenience, targeted to achieve 120 participants over 8 weeks as a pilot study to inform future larger initiatives. Patients that were not responsible for their own medication or those not able to understand questionnaires were excluded.

### Outcomes and Variables Definition

The primary outcome was adherence which was evaluated using the Spanish version of the Morisky Medication Adherence Scale-8 questionnaire that defines low adherence to medications as scores <6 out of 8 points in the scale [17], [18]. This tool has been used extensively to measure adherence in patients with hypertension in primary care settings and has a sensitivity and specificity for identifying low vs. higher adherers of 93% and 53%, respectively [17].

The primary exposure consisted of patients' beliefs towards usage of medication in general as well as their prescribed antihypertensive drugs. These were assessed using the Spanish version of the Beliefs about Medication Questionnaire [19], [20]. This instrument simplifies the broad range of beliefs that individuals hold about pharmacological treatment into four domains: i) ideas of harm: beliefs that medicines are harmful, addictive, poisons which should not be taken continuously; ii) ideas of overuse for general medicines: beliefs that the medicines are overused by physicians; iii) ideas of concern: based on beliefs about the danger of dependence, long-term toxicity and the disruptive effects of medication; and iv) ideas of necessity which assess the individual's beliefs of need for prescribed medication [9], [12], [19].

The first two domains explore beliefs about medication in general, and the latter two about disease-specific conditions, in our case explored about hypertension. Items in each of the domains are rated with a Likert scale from 1 to 5 depending on the level of agreement; higher scores being linked to higher belief levels of harm, overuse, concern or necessity, respectively. Each of the four belief's domains was categorized into low or high, using cutoffs previously established [19]. For harm, concern and necessity domains, scores may range between 5 and 25, considering <15 points as low; whereas scores for the overuse domain may range between 3 and 15 and <9 points was used to define low overuse levels.

Given that the necessity and concern beliefs are therapy-specific, a necessity minus concern differential was calculated as suggested by the BMQ's authors. This differential can serve as a useful approach to identify the relationship between beliefs of necessity and concerns about the same medication in an individual basis i.e. in patient X, necessity beliefs outweigh concerns or vice versa. The scores for the differential may range between −25 and 25 and scores <0, negative values, were considered as low necessity-concern indicating that the patient had more concerns than ideas of need toward antihypertensive medications [19].

Other participant characteristics recorded include age, sex, educational level, length and frequency of administration of anti-hypertensive drugs, and total number of medications taken per day irrespective of their diagnosis.

### Data Collection

Data were collected through a face-to-face encounter. Adherence and beliefs about medications were evaluated using validated questionnaires in Spanish [18], [20]. Each question item was read word by word to each participant and the responses annotated by one of the investigators (MFA).

### Statistical analysis

Categorical variables were analyzed using Chi-square test and Fisher's exact tests. Continuous variables were analyzed using t-test or Mann-Whitney U test, when appropriate. Bivariate and multivariate analyses were performed using a robust variance logistic regression model estimating prevalence ratios (PR) and 95% confidence intervals (95% CI).All statistical analyses were performed using Stata 12.0 (StataCorp, College Station, TX, USA).

### Ethics

All participants were informed of the objectives of the study, and written consent was formally obtained. The study was approved by the ethics committee of HNCH and Brighton and Sussex Medical School Ethics Committee in the United Kingdom.

## Results

A total of 120 participants were recruited. Of these, 5 (4.2%) failed to provide all the data and were subsequently excluded from the analysis. The final sample size of 115 comprised 77 (67%) females, mean age 62.7 years (SD ±11.6).Other baseline characteristics are shown in Table 1.

**Table 1:** Participants' baseline characteristics by adherence group.

	Total	Low adherence	High adherence	p-value*
	n = 115	n = 66	n = 49	
Demographic variables				
Age, mean (SD)	62.7 (11.6)	60.9(11.6)	65.2 (11.4)	0.05
Female, n(%)	77 (67)	47 (61)	30 (39)	0.26
Education level n(%)				
Primary incomplete	11 (9.6)	9 (81.8)	2 (18.2)	0.20
Secondary incomplete	49 (42.6)	28 (57.1)	21 (42.9)	
Secondary complete or more	55 (47.8)	29 (52.7)	26 (47.3)	
Length of treatment n(%)				
Less than 6 months	13 (11.3)	9 (69.2)	4 (30.8)	0.55
More than 6 months	102 (88.7)	57 (55.9)	45 (44.1)	
Number of drugs n(%)				
1–2	55 (47.8)	31 (56.4)	24 (43.6)	0.045
≥3	60 (52.2)	35(58.3)	25 (41.7)	
Number of doses per day n(%)				
1–2	70 (60.9)	41 (58.6)	29 (41.4)	0.10
≥3	45 (39.1)	25 (55.6)	20 (44.4)	

* All p-values calculated with Chi-2 or Fisher's exact test where appropriate.

Low adherence to medications was found in 66/115 (57.4%) subjects. Patients in the lower adherence group were more likely to be younger and to be prescribed more than two drugs than those in the high adherence group. There was no evidence of a difference between low and high adherence groups in terms of sex, educational level, length of treatment and frequency of medication (Table 1).

Overall, there was strong evidence of difference between low (66/115) and high adherence (49/115) groups as per categorization of the Morisky Medication Adherence Scale. For instance, only 1 patient (2%) in the high adherence group acknowledged stopping prescribed medications when out of symptoms, having difficulties in taking all their medicines and feeling hassled about sticking to the treatment plan, whereas >50% of low adherents reported those behaviors. Moreover, more than 80% of low adherents reported to continuously forgetting to take their medicines.

When adherence groups were arranged according to their responses to the Beliefs about Medications Questionnaire, there was evidence of a difference between participants' scores in the harm and concern domains only. In relation to the harm domain, and compared to the high adherence group, mean scores were higher in the low adherence group for the items which state that medicines can do more harm than good, that drugs are toxic, and that drugs can create addiction. The same pattern, higher mean scores in the high adherence relative to the low adherence group, was also observed in the concern domain but no difference in the overuse domain. The opposite pattern, of higher mean scores in the high adherence group, occurred in the necessity domain (Table 2).

**Table 2:** Beliefs about Medications Questionnaire scores by adherence groups.

	Low adherence	High adherence	p-value*
	(mean ± SD)	(mean ± SD)	
**HARM**	**14.3±3.2**	**12±2.5**	**<0.01**
1. People who take medication should stop their treatment for a period of time once in a while.	2.3±1.1	1.9±0.7	0.13*
2. Most of the medicines are addictive	3.3±1.2	2.9±1.1	0.05*
3. Natural remedies are safer than medicines	3.4±1.1	2.7±1	<0.01*
4. Medicines do more harm than good	2.6±0.8	2.1±0.6	<0.01*
5. All medicines are toxic	2.7±1.1	2.4±1	0.13*
**OVERUSE**	**10.8±2.5**	**10.3±2.2**	**0.19** *
1. Doctors prescribe too many medicines	3±1.1	2.8±1.1	0.23*
2. Doctors place too much trust on medicines	3.8±0.9	3.7±0.8	0.25*
3. If doctors spent more time with patients, they would prescribe fewer medicines.	3.9±1.1	3.8±1.2	0.81*
**NECESSITY**	**19.2±4.2**	**20.6±3.7**	**0.10** *
1. Currently, my health depends on my medication	3.8±1	4.2±0.9	0.03*
2. My life would be impossible without medication	3.6±1.2	3.8±1.2	0.18*
3. Without medication I would be very ill	3.9±1	4±1.1	0.29*
4. My health in the future will depend on my medication	3.9±0.9	4.2±0.9	0.08*
5. My medication prevents my condition from worsening	4±0.8	4.4±0.7	0.02*
**CONCERN**	**17.4±3.5**	**14.4±4**	**<0.01** *
1. Having to take my medicines worries me	3.8±1	2.9±1.2	<0.01*
2. I sometimes worry about the long-term effects of my medication	3.9±1	3.2±1.2	<0.01
3.My medicines are a mystery to me	3.6±1	2.8±1.1	<0.01
4. My medication disrupts my life	2.8±1.1	2.3±1	0.03*
5. I sometimes worry about being too dependent on my medicines	3.4±1.1	3.2±1.3	0.41*
**NECESSITY-CONCERN**	**1.8±4.7**	**6.2±4.6**	**<0.01**

*All p-values calculated with t-tests, except in those indicated with an asterisk where U Mann-Whitney was used.

In the multivariable analysis, there was evidence of an association between beliefs related to harm, concern and the necessity-concern differential with adherence groups. In adjusted analyses, and compared to the low harm group, those who had higher harm levels were 52% less likely to be high adherents, while those who had higher concern levels about their medications were 41% less likely to be high adherents when compared with the low concern group. Those in the group with high necessity-concern differential, compared to the low necessity-concern group, were 2.65 times more likely to be adherent to their medications (Table 3).

**Table 3:** Association between patient's beliefs about their medication and adherence levels.

	Crude analysis	Adjusted analysis
	PR (95% CI)	PR (95% CI)
HARM		
Low	1 (Reference)	1 (Reference)
High	**0.45 (0.23–0.89)**	**0.48 (0.25–0.93)**
OVERUSE		
Low	1 (Reference)	1 (Reference)
High	0.76 (0.49–1.17)	0.81 (0.52–1.27)
NECESSITY		
Low	1 (Reference)	1 (Reference)
High	1.42 (0.70–2.86)	1.71 (0.83–3.56)
CONCERN		
Low	1 (Reference)	1 (Reference)
High	**0.56 (0.37–0.87)**	**0.59 (0.39–0.91)**
NECESSITY – CONCERN		
Low	1 (Reference)	1 (Reference)
High	**2.76 (1.3–5.87)**	**2.65 (1.21–5.81)**

All regression models included data from 115 participants and compared low (reference) versus high adherence groups. Bold estimates were significant, p<0.05. Adjustment in the multivariable analysis included age, sex, and length of treatment, number of drugs, and number of doses per day.

## Discussion

Low adherence to antihypertensive medications is very common, reaching approximately 60% in the sample studied. For a population with an established diagnosis of hypertension, and already using health services, these rates indicate a significant problem given all the known complications that are related to hypertension. These results indicate, as the tip of an iceberg, that the problem will likely be worse among those people at the community level, who are aware of their hypertension status but for one reason or another are not using or visiting health services.

Despite the wide variability of adherence rates reported around the world, the adherence rate (42.6%) found in our study was markedly lower than that encountered in other countries such as Colombia (91%), Canada (77%), China (69.2%), Portugal (48.2%), Brazil (51.4%) and only similar to that reported in South Korea (42.4%) [10], [14], [21], [23], [24]. This alarmingly poor adherence is also present in other studies in Peru. Carhuallanqui *et al.* reported a 37.9% of adherence in a study conducted in the same general hospital but using a shorter version of the Morisky Medication Adherence Scale questionnaire [26]. Other local studies described adherence frequencies somewhat higher ranging from 54% to 63% for general hospitals in Trujillo and Lima [27], [28]. Higher adherence levels in these hospitals compared to our findings may be related to differences in the type of health insurance, as these hospitals are part of the social security system and treatment is generally free for their patients.

In the literature, several factors have been related to adherence. For instance, have found old age to be positively associated to this property. In this regard, a study performed in elderly people in New York City, found a low adherence rate of 34.2% [10], [21], [22] while factors that increase treatment complexity, such as the administration of several doses per day or the prescription of multiple medications, have been linked to low adherence [25]. In this regard, we only found the use of several medications to be negatively associated with adherence level. Thus, practitioners should pay special attention and deeply explore the beliefs about medication in this particular prone-to-low-adherence group. Moreover, other interventions, such as telephone-based reminders or alarms, might be considered and implemented in this group as complex prescription may be difficult to follow.

We also found that beliefs about medications play an important role in characterizing adherence patterns. Higher levels in beliefs of drugs' harm and higher concerns about pharmacological hypertensive treatment were related to low adherence after adjustment for confounding factors. On the other hand, beliefs of necessity and overuse did not seem to have an impact on adherence levels. Both, high and low-adherence groups, had high beliefs of necessity and these were the higher scores among all the studied domains while both groups had overuse scores near the cutoff point for high/low overuse beliefs reflecting a neutral position about the excessive prescription of drugs by physicians. However, other studies have linked higher necessity levels and lower concern levels with better adherence levels [22].

Beliefs of general drugs as harmful agents were more commonly present in the low adherence group who strongly considered that medications are toxic compounds and that these can produce more harm than good. It is understandable that patients who share these ideas would be prone to avoid or skip prescribed medications which highlights the importance of exploring and facing patients' ideas of harm during the encounter as an intervention to increase medication adherence.

Regarding the disease-specific sections, low adherents had more concerns about their antihypertensive medications compared to high adherents which was also reported in the United States [29]. These results only reinforce that all patients' concerns, not only about medication but in general, needs to be addressed by their phisicians and that physician-patient communication is an important and simple tool to increase medication adherence, especially in chronic diseases.

An interesting finding was that the differential of necessity minus concern about antihypertensive drugs was found to be a strong predictor of high adherence status, in the order of 2.65 times higher. Therefore, beliefs of necessity must outweigh patients' concerns in an individual to highly adhere to a treatment. Practitioners should take into account that low adherence is likely to develop in a patient with high necessity beliefs if their concerns are higher. Once again, this result remarks the importance of addressing patients' concerns about therapy as a promising tool to promote high adherence among patients with hypertension.

This study expands previous observations by assessing the beliefs and attitudes of patients towards their pharmacological treatment. The instruments used allowed the quantification of qualitative characteristics but also limited the study to the specific domains evaluated by the questionnaire. For instance, aspects such as economic issues that may play a role in medication adherence in patients that cannot afford their prescribed treatment were not considered.

Despite the differences found, we acknowledge that our study may have been underpowered to find larger differences. The target sample size chosen was decided around duration of the fieldwork data collection period. Still, these differences can inform future work in the area and complement the findings of 37% adherence rates described in a previous publication [26]. By selecting the participant population in a hospital setting, the results of this study may overestimate the rate of high adherence as all participants do have a higher degree of awareness of their hypertensive condition compared to the general population.

Previous population-based studies also in Lima have shown poor awareness and control rates of hypertension [6], [30]. Another limitation of our design was not having updated blood pressure records at the interview encounter; a decision made to avoid higher blood pressures related to a white-coat effect. This limited the possibility of linking reported adherence to actual control of blood pressure levels as well as their relationship with medication's belief.

Notoriously, almost all participants rated 4 out of 5 points on the statement that “physicians would prescribe less medication if they spent more time with patients”. To improve current rates of poor adherence, a rational use of medications and a special approach to the patient who is exposed to more than two drugs together with an exploration of necessity and concern about their medications is needed. This interaction would improve the provision of information about drugs' risks and benefits and should clarify all the patient's concerns in a truly shared-decision making process [31], [32].
